# Basics and Frontiers on Pancreatic Cancer for Radiation Oncology: Target Delineation, SBRT, SIB Technique, MRgRT, Particle Therapy, Immunotherapy and Clinical Guidelines

**DOI:** 10.3390/cancers12071729

**Published:** 2020-06-29

**Authors:** Francesco Cellini, Alessandra Arcelli, Nicola Simoni, Luciana Caravatta, Milly Buwenge, Angela Calabrese, Oronzo Brunetti, Domenico Genovesi, Renzo Mazzarotto, Francesco Deodato, Gian Carlo Mattiucci, Nicola Silvestris, Vincenzo Valentini, Alessio Giuseppe Morganti

**Affiliations:** 1 Dipartimento di Diagnostica per Immagini, Radioterapia Oncologica ed Ematologia, UOC di Radioterapia Oncologica, Fondazione Policlinico Universitario “A. Gemelli” IRCCS, 00168 Roma, Italy; francesco.cellini@policlinicogemelli.it (F.C.); giancarlo.mattiucci@policlinicogemelli.it (G.C.M.); vincenzo.valentini@policlinicogemelli.it (V.V.); 2 Radiation Oncology Center, Department of Experimental, Diagnostic and Specialty Medicine—DIMES, University of Bologna, S. Orsola-Malpighi Hospital, 40138 Bologna, Italy; alearceese@hotmail.it (A.A.); mbuwenge@gmail.com (M.B.); amorganti60@gmail.com (A.G.M.); 3 Radiotherapy Unit, Azienda Ospedaliera Universitaria, 37126 Verona, Italy; nicola.simoni@aovr.veneto.it (N.S.); renzo.mazzarotto@aovr.veneto.it (R.M.); 4 Department of Radiation Oncology, SS. Annunziata Hospital, "G. D’Annunzio" University, Via Dei Vestini, 66100 Chieti, Italy; lcaravatta@hotmail.com (L.C.); d.genovesi@unich.it (D.G.); 5 Radiology Unit—IRCCS Istituto Tumori “Giovanni Paolo II” of Bari, 70124 Bari, Italy; acalabrese22@gmail.com; 6 Medical Oncology Unit, IRCCS Istituto Tumori “Giovanni Paolo II”, Viale Orazio Flacco, 65, 70124 Bari, Italy; dr.oronzo.brunetti@tiscali.it; 7 Department of Neuroscience, Imaging and Clinical Sciences, “G. D’Annunzio” University, 66100 Chieti, Italy; 8 Istituto di Radiologia, Università Cattolica del Sacro Cuore, 00168 Roma, Italy; francesco.deodato@gemellimolise.it; 9 Radiotherapy Unit, Gemelli Molise Hospital, 86100 Campobasso, Italy; 10 IRCCS Istituto Tumori “Giovanni Paolo II” of Bari, 70124 Bari, Italy; 11 Department of Biomedical Sciences and Human Oncology, University of Bari “Aldo Moro”, 70124 Bari, Italy

**Keywords:** pancreatic cancer, radiotherapy, immunotherapy, SBRT, guidelines, review, CTV, MR guided RT, protons, SIB

## Abstract

Pancreatic cancer represents a modern oncological urgency. Its management is aimed to both distal and local disease control. Resectability is the cornerstone of treatment aim. It influences the clinical presentation’s definitions as up-front resectable, borderline resectable and locally advanced (unresectable). The main treatment categories are neoadjuvant (preoperative), definitive and adjuvant (postoperative). This review will focus on (i) the current indications by the available national and international guidelines; (ii) the current standard indications for target volume delineation in radiotherapy (RT); (iii) the emerging modern technologies (including particle therapy and Magnetic Resonance [MR]-guided-RT); (iv) stereotactic body radiotherapy (SBRT), as the most promising technical delivery application of RT in this framework; (v) a particularly promising dose delivery technique called simultaneous integrated boost (SIB); and (vi) a multimodal integration opportunity: the combination of RT with immunotherapy.

## 1. Introduction 

Pancreatic ductal adenocarcinoma (PDAC) will potentially represent the second leading cause of cancer death by 2030. The 5-year overall survival (OS) rate is approximately 7–10% [1,2]. Even under the most optimal clinical trial conditions, the median survival of resected patients following adjuvant therapy ranges from 20 to 28 months [3,4,5,6]. Locoregional failure is expected to affect 50–80% of patients; the systemic relapse (locoregionally associated or not) will affect over 70% of patients, leading to a specific 5-year survival rate around 10–20%, even for radically resected patients [7]. Interestingly, among patients with relapse and death, 30% will only experience locoregional disease progression [8]. 

In summary, pancreatic cancer represents a modern oncological urgency; its management takes both a distal and local approach. 

### 1.1. Clinical Presentation

Resectability is a crucial goal for pancreatic cancer treatment. From this perspective, the main clinical presentations can be grouped into three scenarios according to tumor involvement of the adjacent vasculature (and thus the expected probability of resectability): first, the up-front resectable (to which the management of adjuvant postoperative settings also refers); second, the locally advanced pancreatic cancer (LAPC), which is inoperable at first presentation; and third, the borderline resectable pancreatic cancer (BRPC), in which resection is technically possible albeit assuming a high risk of a positive margin and therefore is at increased risk of recurrence [9]. If the treatment cornerstone is represented by surgery, providing microscopically free-margin resection (R0) is currently the only way to obtain the best possible cure rate [10]. Macroscopical (R2) and microscopical (R1) margin infiltration have survival trends similar to locally advanced or metastatic presentations [11,12,13,14,15]. For instance, R1 resections have been found to have comparable outcomes to definitive radiochemotherapy (RTCT) without surgery [3,16]. In this regard, a recent retrospective analysis by a national cancer database among 44,852 PDAC patients resected between 2004 and 2013, associated with either neoadjuvant or adjuvant therapy, revealed margin status as an independent prognosticator. The median survival for patients who did not undergo surgery was 10.3 months, compared with 19.7 months for R0 (*p* < 0.001), 14.3 months for R1 (*p* < 0.001) and 9.8 months (*p* = 0.07) for R2 resections [17]. 

Unfortunately, at first diagnosis, only 10–20% of PDAC patients present primarily resectable disease and more often have microscopically positive margins at the time of surgery [7,9,18,19]. The issue of the expected rate of microscopically negative resections for up-front resectable presentations should be pointed out. Currently, the criteria defining up-front resectable presentation have still not been unequivocally determined. On the other hand, preoperative diagnostics have some limitations; thus, not all up-front-operated patients can be actually resected. Moreover, not all resected patients (although classified as up-front resectable presentations) will obtain an R0 resection. A systematic review and meta-analysis by Versteijne et al. [20] pooled the results from 12 selected trials on up-front surgery (five prospective, five retrospective and two randomized controlled trials, accounting for 1746 patients) and showed that, among patients with (up-front) resectable pancreatic cancer, the resection rate was 76.8% (95% confidence interval (CI) = 73.8–79.7) and the R0 resection rate was 71.4% after surgery.

In summary, resectability is the cornerstone of treatment. It influences the clinical presentation (resectable, BRPC or LAPC). The ultimate goal is an R0 resection. Unfortunately, the R0 resection rate is suboptimal even for up-front resectable presentations that are therefore suitable for adjuvant or neoadjuvant integrative treatments.

Available treatment options could be shuffled in light of the aforementioned considerations, excluding treatments for palliative, metastatic or recurrent presentations (initially treated or not). The main categories are *neoadjuvant* (preoperative), *definitive* and *adjuvant* (postoperative).

### 1.2. Neoadjuvant Therapy

The neoadjuvant approach is appropriate for LAPC in order to allow conversion to resectability and, for BRPC, in order to increase the chances of an R0 resection. 

In fact, some evidence suggests that an initially unresectable presentation, if converted to operable after neoadjuvant treatment and having undergone microscopically complete resection (R0), obtains similar survival rates to those observed for up-front resectable pancreatic cancer [21]. 

The neoadjuvant approach can be applied to up-front resectable presentations in order (i) to reduce the rate of unresected patients (reported in the literature in spite of presurgical resectability-assigned status), (ii) to further decrease the non-microscopically radical resection rates [20] and (iii) to possibly increase survival outcomes as for other primary diseases (e.g., rectal cancer [22,23,24] or gastro-esophageal cancer [25,26]. As for doubt about delaying surgery for up-front resectable or doubtfully operable (BRPC) patients, some authors have highlighted that patients developing distant metastases through the neoadjuvant course would have probably been at risk of this during the postoperative phase anyway; thus, the neoadjuvant approach can also be exploited to gauge the intrinsic biological aggressiveness of the disease [27].

The neoadjuvant approach usually includes chemotherapy (CT), chemosensibilization concomitant to long course conventionally fractionated radiotherapy (RTCT) and stereotactic body radiotherapy (high-precision hypofractionated radiotherapy without chemosensibilization; SBRT). 

### 1.3. Definitive Therapy

The definitive approach for CT (for distal and/or local progression) or for either RTCT or SBRT (often sequentially associated to CT) is recommended for those LAPCs not expected to convert to resectable. It should be highlighted that, for RTCT or SBRT, the dose delivered with definitive intent might be higher than the dose applied to possibly induce resection, even within this subset of LAPC.

### 1.4. Adjuvant Therapy

The adjuvant approach is usually applied after surgery for up-front resectable patients. It aims to further improve the chances of cure for an R0 resected presentation or to compensate for R1/R2 or doubtful complete resections. Usually, CT is administered in the former while RTCT is currently an option for the latter scenario (SBRT is not routinely applied in this setting). 

### 1.5. Clinical Presentation: Closing Remarks

In summary, neoadjuvant, definitive or adjuvant approaches integrating CT, RTCT or SBRT in a concomitant or sequential modality can be administered to deal with the main non-metastatic clinical presentations (up-front resectable, LAPC and BRPC).

Definitive evidence on the most effective treatment option for each presentation is still lacking due to weak and sometimes contradictory results. Precision oncological treatments for each approach are the solution to this problem but are still yet to be routinely applied. Multimodal integration of surgery with systemic and radio-oncological therapies will probably provide more fruitful opportunities. Modern radiotherapy (RT) is being supported by new technologies and treatment modalities that are rapidly revolutionizing this field. Beside the clinical governance of radiation-oncological principles, the specific core of technical performance in RT is centered on three pillars: dose prescription (i.e., defining the best dose for each clinical presentation with an optimal balance between efficacy and toxicity), target delineation (and its indications per clinical presentation) and dose delivery (practically focusing on the target area, possibly managing both its intra-fraction motions and daily anatomical modifications). 

Thus, we will now focus on (i) current indications of national and international guidelines; (ii) current standard indications for target volume delineation in RT; (iii) emerging modern technologies (including particle therapy and MR-guided-RT); (iv) SBRT, as the most promising technical delivery application of RT in this framework; (v) a particularly promising dose delivery technique called “simultaneous integrated boost” (SIB); and (vi) an upcoming multimodal integrative approach: the combination of RT with immunotherapy.

## 2. Overview of Guidelines and Literature Highlights 

### 2.1. Methodology for the Review of the Guidelines

Guidelines (GL) are formed by the summation of available evidence and help to guide clinicians’ choices. Guideline indications represent the most influential available indications and are also usually advocated in cases of contention regarding clinical practice selection or administration. Even a published phase 3 randomized controlled trial should be mentioned in a GL to be formally considered as a gold standard. We searched for available GLs published online between 2013 and 2020. International and national GLs were included both if endorsed by a specific oncological society (e.g., medical oncological or radiation oncological) or a multidisciplinary network (e.g., the National Comprehensive Cancer Network (NCCN)). The search was restricted to publications in English. We used the following medical subject heading (MeSH) keywords: “pancreatic cancer”, “guideline”, and “management”. The full Medline search term was (“pancreatic neoplasms” (MeSH Terms) OR (“pancreatic” (All Fields) AND “neoplasms” (All Fields)) OR “pancreatic neoplasms” (All Fields) OR (“pancreatic” (All Fields) AND “cancer” (All Fields)) OR “pancreatic cancer” (All Fields)) AND (“guideline” (Publication Type) OR “guidelines as topic” (MeSH Terms) OR “guideline” (All Fields)).

In total, 13 GLs were identified. Five of them were international [3,28,29,30,31,32]. American Society of Clinical Oncology (ASCO) published two separate GLs for resectable and LAPC; two of these were established by multidisciplinary networks, [3,32] while the other three were established by either medical [28,29] or radio-oncological [31] societies. Among the eight national GLs [33,34,35,36,37,38,39,40], one reports the results of a consensus conference [38] and one only deals with LAPC [40]. Of the total, eight were published or updated (including online updates) between 2018 and 2020. Table 1, Table 2 and Table 3 summarize the indications provided by the GL for (up-front) resectable, LAPC and BRPC presentations, respectively. The summary separately reports which option is indicated as the main option (deduced by the GL main text, tables or additional material), separated by the heading “alternative option”.

### 2.2. Resectable Pancreatic Cancer

Twelve of the GLs apply to (up-front) resectable presentation. One of the GLs indicates that enrolling patients into clinical controlled trials (CCT) is the preferable option [3]. For routine clinical scenarios, all the 12 GLs advocate adjuvant CT as the primary option. Among them, eight indicate adjuvant CT as the only approach while four include suggestions for evaluation of adjuvant RTCT (adjRTCT) [3,31,33,39].

Regarding CT regimen preference, the indications were as follows: FOLFIRINOX in six, gemcitabine in two, 5-Fluoruracil (5-Fu) in two and gemcitabine-capecitabine in one of the GL, with S-1 only suggested in the Japanese GL (of note, some of the GLs indicated multiple primary alternatives).

The neoadjuvant approach is only proposed within a CCT by six of the 12 GLs. The other five mention it as a general alternative option for selected patients [3,29,31,38,39], but only two provide any details: CT only [39] and either CT or RT [38], respectively. One of the GLs does not mention this approach at all [28].

Part of the reason for the limited inclusion of routine evaluation of adjRTCT among the treatment options is the contradictory nature of the reported survival endpoints, along with the suspected detrimental effects of adjRTCT administration [43,44]. One of the most well-known studies in this regard is the “ESPAC-1 trial”. In this trial, Neoptolemos et al. randomized 289 patients into four treatment groups (surgery alone, adjCT, adjRTCT and adjRTCT followed by adjCT). They neither found benefit to local control nor to overall survival of the patients receiving adjRTCT; moreover, the integration with RT was detrimental to survival [45]. This trial had several limitations. Some were general, such as the poor adherence to therapy and the low accrual. Some were specifically related to RT, such as the extremely poor level of technology provided and the very low RT dose administered. Moreover, conformal RT planning was not adopted (old “Anteroposterior-Posteroanterior”(AP-PA) an imprecise and more toxic approach, was instead applied); an unusual split course regimens was applied; and a bolus instead of protracted 5-Fu infusion for chemosensibilization of RT was applied [46,47,48]. Due to its inadequate setting, the ESPAC trial must be handled with caution when cited or included in a meta-analysis to draw conclusions about adjRTCT. Given the possibly contradictory results of adjRTCT improving OS, the most relevant evidence derived from the “ESPAC-1” trial is the uselessness of inadequate RT, particularly if not modern and providing inadequate doses. Between 1998 and 2002, the randomized trial RTOG 9704 compared 451 patients with complete gross total resection receiving adjCT (5-Fu vs. gemcitabine) for 3 weeks prior to and for 12 weeks after RTCT (50.4 Gy plus 5-Fu in continued infusion). In multivariate analysis, the effect of gemcitabine treatment was better than that of 5-Fu (*p* = 0.05) and median survival was 20.5 months vs. 16.9 months, respectively. This trial applied modern RT and prospective quality assurance. Interestingly, although data are of course not directly comparable, some features of RTOG9704 can be highlighted with respect to other landmark randomized trials. Compared to the “ESAPC-1 trial”, RTOG9704 resulted in longer survival although having enrolled more unfavorable presentations (by resection status, pN-status and tumor size) [48]. Moreover, in the subgroup analysis (by serum CA19-9 levels of ≤90 vs. >90 U/mL and the RT protocol) reported by Berger et al., comparing 5-year survival results to those of the CONKO-001 trial (a landmark trial assessing the role of adjCT over surgery alone), RTOG9704 had a favorable survival outcome, with a median OS of 24 vs. 22.1 months and a 5-year OS of 34 vs. 21% (despite an R1 resection rate of 35% in RTOG9704 and 17% in CONKO-001) [47,49]. We are of course far from hypothesizing that adjRTCT could be superior or preferable to adjCT on the basis of these findings, but they at least question the role of inadequate RT in providing consistent and reliable evidence. The administration of modern RT (i.e., with modern conformal techniques and at sufficiently high doses) impacts both efficacy and toxicity, possibly affecting the clinical outcome. Hsu et al. reported the survival benefit of adjRTCT over surgery alone in their retrospective analysis of 1092 patients treated between 1985 and 2005 (up to 50.4 Gy using a modern technique) in two centers [50]. With a median survival of 18.8 months, the matched-pair analysis by treatment group found longer OS with RTCT: median survival 21.9 vs. 14.3 months, and 2- and 5-year OS 45.5 vs. 31.4% and 25.4 vs. 12.2%, respectively (*p* < 0.001). More recently, a multicenter retrospective review by Morganti et al. involved 955 consecutive patients who underwent to R0-1 resection (T1-4, N0-1 and M0) treated between 1995 and 2008 with the application of modern RT techniques [51]. Among the analyzed patients, 623 received postoperative RT, 575 received RTCT and 462 received adjuvant CT. Median follow-up was 21.0 months. The reported median OS for the group having received adjuvant RTCT was 39.9 months, significantly different from the group of patients not having received adjuvant RTCT (*p* < 0.001). The issue of the RT-delivered dose is even more crucial. Morganti and colleagues performed a multicentric retrospective analysis on 514 patients with PDAC (T1-4, N0-1 and M0) treated with R0-R1 surgical resection followed by adjuvant RTCT [52]. With a median follow-up of 35 months (range 3–120 months), they highlighted the significant impact on OS by higher RT dose ranges vs. much lower ones, confirmed by multivariable analysis. In particular, doses below 45 Gy (similar but still higher than in the ESPAC trial) had the lowest survival (13 months). Conversely, progressively higher dose ranges were associated with proportional survival improvements: 21 months for ≥45 and <50 Gy, 22 months for ≥50 and <55 Gy, and 28 months for ≥55 Gy (*p* = 0.004). This evidence suggests that the conflicting results of randomized trials on adjuvant RTCT in PDAC could be due to the <45 Gy dose used and the inadequate technologies applied. Therefore, further studies are justified in this field, which combine advanced RT techniques with the standard CT regimen in the adjuvant setting (FOLFIRINOX), as defined by the “PRODIGE” Group’s study [53]. Moreover, the final results of RTOG0848, comparing RT + 5FU or capecitabine after CT, to CT alone will further clarify the issue of combining RTCT to CT in resected pancreatic cancer (PC) [54].

### 2.3. Locally Advanced Pancreatic Cancer (LAPC)

Thirteen of the GLs deal with LAPC. One of the GLs indicates that enrolling patients into CCT is the preferred option [3]. For routine clinical scenarios, all of the GLs include CT as the primary option (one of the GLs among this subgroup specifies that such indication is technically based on very low-quality data) [39]; while two also include RT as a possible primary option [3,37]. Nine of the GLs indicate that induction CT possibly integrated with either RTCT or SBRT while two exclusively include CT in the treatment flow [35,40], unless treatment fails after the entire CT course [40]. Indications on reassessment for response (possibly adding RTCT to the CT-only approach) ranges from 3 to 6 months.

With respect to the preferable CT regimen, the GL indications were as follows: FOLFIRINOX was indicated in eight, gemcitabine in six, gemcitabine-nab-paclitaxel in four, gemcitabine-abraxane in two [39,40], and PEXG/PAXG in two of the GLs [39,40] (of note, some GLs indicated multiple primary alternatives). Two of the GLs indicated that there is no clear evidence to support one regimen over another [30,35].

With respect to RT details, the GL proposed RTCT as definitive or in a sequential integration with CT. With respect to SBRT, three GLs indicate SBRT as a possible clinical option to the main treatment flow [3,30,31], one indicates it as a possible salvage therapy after failure of the complete CT course [40], and one indicates it as an option reserved to CCT [38].

One of the most relevant, modern, randomized trials having influenced treatment for LAPC was the LAP-07 trial [55]. The randomized trial of Hammel and colleagues had two levels of randomization: the first was regarding CT administration of gemcitabine alone vs. combined with erlotinib (449 patients) and the second was regarding the use of gemcitabine vs. RTCT (54 Gy, with concomitant capecitabine) in 269 patients. The trial did not find any significant survival benefits for RTCT over CT for median OS (primary endpoint; CT, 16.5 months vs. RTCT, 15.2 months; *p* = 0.83) or disease-free survival (secondary endpoint; CT, 8.4 months vs. RTCT, 9.9 months; *p* = 0.06). Significant differences favoring RTCT were conversely found for other secondary endpoints: locoregional progression (RTCT, 32% vs. CT, 46%; *p* = 0.04) and for the time to resumption therapy with second-line CT (RTCT, 6.1 months vs. CT, 3.7 months). Metastatic progression significantly favored CT (44%) over RTCT (60%, *p* = 0.04). Although the results of the LAP-07 trial discourage the inclusion of RTCT in the routine treatment for LAPC [56], the paper from Hammel et al. discusses balancing the role of RTCT by “confirming the safety of RTCT (with concurrent capecitabine)” and advocating the “need for further RT intensification”, in particular, intensity-modulated RT (IMRT) and SBRT for the safe escalation of doses [55]. Again, the role of the RT-administered dose is of importance. Krishnan et al. reported on 200 LAPC patients undergoing induction of CT followed by RTCT between 2006 and 2014, applying modern RT [57]. Among them, 24% was selected for dose-escalated IMRT with a biologically effective dose (BED) over 70 Gy, while the median radiation dose delivered to the entire cohort was 50.4 Gy (BED = 59.47 Gy). Patients who received BED > 70 Gy had a superior OS (17.8 vs. 15.0 months, *p* = 0.03), which was preserved throughout the follow-up period. Estimated OS rates at 2 years were 36 vs. 19% and at 3 years were 31 vs. 9%. BED > 70 Gy also improved local-regional recurrence free survival (10.2 vs. 6.2 months, *p* = 0.05) compared to those receiving BED ≤ 70 Gy. The role of technology providing safe and more efficient RT is interestingly highlighted in a paper by Colbert and colleagues [58]. In their study, IMRT was applied to dose-escalate RT (up to BED > 70 Gy using three different schedules) in 39% of a global cohort of 154 LAPC patients. For patients receiving dose escalation, technological support for daily dose delivery was also applied (including daily image guidance and breath-holding techniques). The rate of patients who experienced no acute toxicity was higher in the BED > 70 Gy group than the standard group (36 vs. 15%, *p* = 0.001). Moreover, for patients treated with BED > 70 Gy IMRT, a lower risk of acute toxicity was associated with a later treatment year (*p* = 0.007), stressing the link between modern technology and clinical opportunities. SBRT represents one of the most promising opportunities for LAPC. The role of SBRT will be extensively discussed in the following sections.

Finally, it should be considered that some GLs, such as those by the NCCN, ESMO, ASCO and ASTRO, include RT in the palliative treatment of metastatic and non-metastatic locally advanced PC with the aim of relieving pain, bleeding or obstructive symptoms. These GLs do not recommend a specific RT regimen but suggest a personalized approach based on patient and tumor characteristics [3,28,30,31]. Among the few evidence on pain control achieved with RT in this setting, we can report a 75% rate of pain relief recorded in a small series of patients treated with 30 Gy in 10 fractions with standard techniques [59] and an 85% pooled response rate after SBRT, as reported in a systematic review [60].

### 2.4. Borderline Resectable Pancreatic Cancer (BRPC)

Ten of the GLs address BRPC presentations. Four of the GLs indicate the preferable option is to enroll patients into CCTs [28,34,35,37], all but one of which [35] also provide indications for routine clinical scenarios. For routine clinical scenarios, nine of the GLs indicate neoadjuvant therapy as the primary option. Neoadjuvant therapy is detailed as CT in two GLs [33,39], up-front RTCT is detailed in two GLs [3,33] and induction CT possibly followed by RT is detailed in six of the GLs (of note, some GLs indicated multiple primary alternatives). In particular, SBRT is indicated as evaluable in clinical practice for BRPC by only one of the GLs [31], while one other mentions it as evaluable within CCT [38].

Anatomical criteria define the level of tumor abruption into vessels [3], as initially proposed by Kats et al. of the MDAnderson Group [27]. Some of the currently accepted older reports do not specifically refer to the same type of borderline presentation; thus, all studies are not easily compared. The efficacy of neoadjuvant therapy over surgery alone can be underlined by the recent meta-analysis of Versteijne and coworkers [20]. This comprised 38 studies, including 3484 patients, 49.9% of whom received neoadjuvant treatment (including both CT and RT). The analysis included both up-front resectable and BRPC series (16 resectable, 18 BRPCs and four mixed). The weighted median OS after neoadjuvant treatment in 881 patients with BRPC was 19.2 months (range 11–32 months) vs. 12.8 months (range 11.6–16.3 months) after up-front surgery (for 927 BRPC patients). The R0 rate was significantly higher for neoadjuvant treatment than up-front surgery: 86.8% (95% CI: 84.6–88.7) vs. 66.9% (95% CI: 64.2–69.6; *p* < 0.001). In total, 17.8% of patients (306 patients among the 29 out of 35 trials reporting these data) did not proceed to exploratory surgery. Disease progression (either local or distant metastasis) was the most common reason for this (64.4% of patients).

In a subgroup analysis of the meta-analysis by Versteijne et al. [20], RT vs. CT among the studies administering neoadjuvant therapy (six CTs, 24 RTCTs and five mixed) were compared. The authors stated that the results were inconclusive and “to be interpreted with caution”. The weighted median OS was 20.9 months (range 13.6–27.2 months) and, for patients who received CT alone, 17.8 months (range 9.4–32 months). Besides the still open issue of best efficacy, due to the high rate of metastatic development, the interest in administration of systemic therapy before RT is growing [61]. Efficacy of RT over surgery alone has been addressed in various studies, including some retrospective and prospective randomized trials [62]. Usually the older and more recent series were applied: RTCT long-course treatment (doses ranging close to 50 Gy) or slightly hypofractionated schedules (30 Gy -3 Gy per fraction (Gy/fx); 30 Gy -2Gy/fx; and 36 Gy -2.4Gy/fx) [61,63]. Most modern series apply SBRT, as this avoids the potential disadvantages of longer courses, reduces time to surgery, shortens the treatment duration for the patient and provides higher biologically equivalent doses to the tumor in a shorter time and, of particular interest, can be more easily overlapped with CT. Moreover, the preliminary results of the PREOPANC-1 trial showed that preoperative CRT significantly improves outcome in BRPC compared to up-front surgery [64].

### 2.5. Closing Remarks

In summary, CT is generally the primary indicated approach, both in neoadjuvant and adjuvant settings, for resectable presentations, LAPC and BRPC. The number of GLs recommending RT rises from one third for resectable presentations to over two thirds for both LAPC and BRPC; combining RT with CT is mostly (but not only) indicated into a sequential approach; time to reevaluations after CT administrations varies from a minimum of 3 to a maximum of 6 months; the neoadjuvant approach is mostly suggested within CCT for resectable presentations while also mostly suggested for BRPC; SBRT is specifically indicated by some of the GLs as an available clinical option for LAPC, while RTCT is currently preferred for BRPC in clinical practice.

This overview suggests that, notwithstanding the firm current indication for CT, there is a need for new and efficient clinical options. Some of the GLs suggest that the treatments currently applied are not the best but rather the best that are currently available. In fact, the specific grade of indication for referring patients to CCT (mostly for BRPC), the specific indications regarding the low-quality level of data supporting CT for LAPC [39], the indication that three to five different main CT schedules are equally applicable, along with specific comments regarding the impossibility of choosing one option over another in terms of efficacy (particularly in a modern precision medicine scenario) and the varying level of involvement of RT in the treatment workflow indicated by various GL suggest the urgency of identifying new single or multimodal integrated treatment opportunities. Given the proven efficacy of CT for distant metastases control and RT for locoregional control [55], seeking new modalities of integration allowing for the shortest discontinuation of CT (such as SBRT; see the following paragraphs) would probably be very helpful.

## 3. RT Schedules

A brief summary of the international radio-oncological schedules according to the ASTRO guidelines [31] and NCCN [3] is outlined herein.

For adjuvant settings, RTCT doses ranging from 45–54 Gy with daily fractions ranging from 1.8–2.0 Gy are recommended by ASTRO, with concomitant chemosensibilization by 5-Fu [31]. The NCCN specifies that a 5–9 Gy boost over the generally administered dose of 45–46 Gy can be applied to the tumor bed and anastomoses, if clinically appropriate [3]. Doses higher than 54 Gy should be avoided outside a CCT.

For LAPC, RTCT is recommended at doses ranging from 45–54 Gy (at 1.8–2.0 Gy/fx) [3] or 54–56 Gy with daily fractions ranging from 1.75–2.2 Gy [31]. SBRT is recommended by ASTRO at doses ranging from 33–40 Gy with daily fractions of 6.6 or 8 Gy [31]. The NCCN reports 30–45 Gy for treatment in three fractions or 25–45 Gy for treatment in five fractions [3].

For BRPC, ASTRO recommends RTCT at doses ranging 45–50 Gy with daily fractions ranging from 1.8–2.0 Gy. The NCCN reports 45–54 Gy (1.8–2.0 Gy daily) or a schedule at 36 Gy with 2.4 Gy per fraction [3]. SBRT is recommended by ASRTO at doses ranging from 30–36 Gy with daily fractions of 6.0–6.6 Gy. If an SIB technique (see the in-depth discussion in the following sections) is adopted, the two dose levels include doses ranging from 30–36 Gy with daily fractions of 6.0–6.6 or 8 Gy to the gross tumor volume (GTV) and a simultaneous dose of 40 Gy with daily fractions of 8 Gy to the vessels. The NCCN does not address indications in this framework.

## 4. Target Volume Delineation

### 4.1. Conventional Radiotherapy

Data has shown that the technical quality and administration of radiation therapy (RT) or deviations from established quality assurance guidelines have had a relevant impact on clinical outcomes and that standardized atlases for RT and case examples could improve protocol treatment compliance [65]. In this regard, the Radiation Therapy Oncology Group provided a consensus panel GL for the delineation of the clinical target volume (CTV) in the postoperative treatment of pancreatic head cancer [66].

High-resolution dual-phase contrast enhanced Computed Tomography scan (CTscan) represents the primary modality for PDAC staging, detecting vascular invasion and defining resectability criteria and is the predominant imaging modality used for pancreatic tumor delineation in RT planning. Magnetic resonance imaging (MRI) could be considered for RT planning, improving delineation accuracy due to the high resolution of soft tissue. Furthermore, MRI is able to detect changes in tumor size and normal organ position over the course of RT. For this reason, MRI is emerging as a component of adaptive real-time strategies with on-board MRI guidance [67].

Recently, recommendations for GTV delineation of PDAC using MRI have been provided and their use is advised [68].

Furthermore, a report by an international contouring symposium of expert gastrointestinal radiation oncologists reported a smaller GTV defined on MRI compared with CTscan. A stepwise method for GTV delineation when using abdominal MRI was proposed [69].

Given the increasing relevance of MRI in the context of pancreatic cancer RT, a multicenter contouring study was also conducted by the study group for gastrointestinal cancers of the Italian Association of Radiation Oncologists (AIRO), aiming to evaluate the impact of diagnostic MRI in GTV delineation for BRPC and LAPC. This study confirmed that a smaller GTV is delineated on MRI compared with CTscan in the case of BRPC. On the other hand, a large contouring variability was reported in LAPC cases, suggesting that the tumor area close to vascular structures or involving them should be considered as a region of complex anatomy for GTV delineation, requiring very accurate guidelines for volume definition and confirming the results of previous studies [70,71] and as also highlighted by the expert panel of the international contouring conference [69].

Although there is no consensus concerning the elective nodal irradiation (ENI) in pancreatic cancer RT, it could be justified in treatment with curative intent. Some indications for the CTV definition of ENI treatment have been given in the past in relation to the treatment of head pancreatic carcinoma [72,73]. More recently, based on a review of 18 pathologic reports [74], the high risk lymph node regions related to the primary tumor site (head or body/tail of pancreas) were proposed as ENI areas and an atlas reporting standard criteria for the CTV definition and delineation in the preoperative or exclusive treatment of PDAC was created [75].

### 4.2. Stereotactic Body Radiation Therapy (SBRT)

The Australasian Gastrointestinal Trials Group (AGITG) and Trans-Tasman Radiation Oncology Group proposed a workshop to standardize dose, simulation technique, and volume delineation for CTV for SBRT of PC. Consensus was achieved, and a contouring atlas was published as a guideline [76].

Panels strongly encourage treatment with an active breath hold or gated technology. In patients treated during free breathing, an internal target volume should be delineated using a 4-dimensional computed tomography (4DCT scan) or 4DMRI, and if tumor or fiducial movement is greater than 5 mm, amplitude-reducing methods, such as gating, tracking, compression, or a combination, are recommended.

During simulation, a contrast-enhanced CT scan should be performed for increased reproducibility of GTV delineation [69]. All available diagnostic images, including CT, MRI and positron emission tomography (PET)-CT, should be ideally performed in the treatment position and may be fused to further assess organ motion and to delineate target volume.

In aiming to standardize volumes delineation, clear definitions were provided by the panels for the primary GTV and tumor-vessel interface (TVI). The GTV should include fibrotic areas near and at the vessels in case of suspected tumor involvement.

The TVI is recognized, even in previous studies [77,78], as the area involved in or in close proximity to major vessels (including the celiac artery, superior mesenteric artery, common hepatic artery, left gastric artery, superior mesenteric vein, portal vein, splenic vein or aorta) and the GTV, where recurrences and close margins commonly occur. Stepwise instructions for GTV and TVI delineation have been provided. The GTV and nearby vessels are contoured. A 5-mm expansion of the GTV helps delineate which vessels are within 5 mm of the GTV. The entire circumference of the involved or proximal vessels is contoured to outline the TVI. The GTV and TVI are combined to obtain the CTV.

The panel does not recommend elective nodal volumes in the absence of prospective data for SBRT of PC.

Finally, when highly conforming and dose-gradient RT techniques are used, an accurate definition of the organ-at-risk (OAR) is mandatory following an available contouring atlas [68].

## 5. Future Directions

### 5.1. Particle Therapy

Particle therapy is emerging as a promising option for pancreatic cancer patients. Proton therapy (PT) has a potential benefit over photons by traversing a finite distance into tissue, releasing the majority of its energy in a narrow site defined as the “Bragg Peak”, with no exit dose beyond the target [79].

These properties can theoretically allow for dose reduction to OARs (minimizing potential toxicities), simultaneously delivering higher doses to the tumor [80]. In a recent dose-escalation study, LAPC patients treated with PT to a dose of 54.0–67.5 GyE (Gray equivalents) in 25–33 fractions experienced an improvement in local control (LC) and OS compared to previous photon data, with negligible toxicity [81]. Moreover, PT has shown promising results as a neoadjuvant treatment with favorable results in terms of resection rate and survival [82]. In addition to the aforementioned “Bragg Peak”, carbon-ion radiotherapy (CIRT) exhibits a superior relative biological effectiveness (RBE) due to higher linear energy transfer (LET) compared to photons and protons, being able to potentially overcome the intrinsic radioresistance of pancreatic tumors [83]. A phase I trial evaluated the safety and efficacy of CIRT for pancreatic cancer in a neoadjuvant setting. Twenty-six patients were enrolled, and 21 (81%) underwent surgery. The 5-year survival rate was 42%, with no patients experiencing local recurrence [84]. The retrospective multi-institutional J-CROS Study 1403, evaluating 72 patients with LAPC treated with CIRT to a dose of 52.8–55.2 GyE in 12 fractions, showed a median OS of 21.5 months with a 2-year local recurrence rate of 24% [85].

### 5.2. MR-guided-RT (MRgRT)

MRI is useful for diagnostics in pancreatic cancer: similarly, it has also become useful for RT administration. The concept of MR guidance on RT refers to various different issues, including the information added to the contouring of target volumes. Heerkens and colleagu*es* have described how multiparametric MR pretreatment scanning could improve the conventional Linac-based SBRT administration [86]. They defined target and OAR motion by the registration of four-dimensional (4D) CTscan simulations with both contrast-enhanced CT scan and MR (using a 1.5 Tesla scanner). Using MR two-dimensional cine acquisition, the peak-to-peak motion in the craniocaudal and anteroposterior directions were calculated. After treatment planning, the static dose distribution was convolved with the cine MRI-based motion trajectory to simulate the delivered dose to the tumor and OARs. Twenty patients were treated with SBRT (24 Gy/8Gyfx). No grade 3 or higher treatment related toxicity was observed. One of the most interesting approaches of MR integration in RT is represented by MR-hybrid machines. Linac accelerators with integrated MR scanners provide such aid through the process of simulation, planning and delivery: the so-called MR-guided RT (MRgRT). Unity (Elekta, Stockholm, Sweden) uses a 1.5 Tesla MRI scanner with a 7 MV Flattening Filter Free (FFF) Linac; the MRIdian system (ViewRay, Cleveland, Ohio) applies a 0.35 Tesla MRI scanner with a 6 MV FFF Linac [87].

Currently, such systems administer RT though IMRT by the step-and-shoot approach and still cannot perform more complex modulation arrangements like sliding windows IMRT and volumetric modulated arc radiotherapy (VMAT), which is particularly useful for the optimum arranging of dose distribution. Nevertheless, the better on-board-image guidance provided by MR vs. the cone-beam CT (CBCT) and the chance to apply MR gating to treatment sessions has exclusive advantages for daily individuation of the target and OAR. The MRIdian system is currently the most widely clinically tested and available. One peculiarity of this system is live treatment gating of both target and OARs through the entire treatment session by direct and fiducial-less visualization of the structures of interest. The gating can be highly personalized, fraction-by-fraction. Treatment gating protocols can be directly applied to target volumes, to surrogate target volumes (especially if the target is not clearly visible on positioning images) or even to OARs in order to optimize their sparing [88,89].

Finally, the most important benefit is the ability to perform “on-line adaptive RT”. Targets and OARs are recontoured before the RT session while the patient lays on the treatment couch, obtaining an “adapted” prediction of the dose distribution of the day (taking into account the occurred anatomical variations), and if needed, an optimized plan can be reloaded and then delivered. All these potential advantages can be exploited to prescribe a higher biological dose while avoiding undue high doses to adjacent critical organs such as the duodenum, stomach and bowel [90].

There a still few clinical reports available: Henke et al. included five patients (two LAPC and one recurrent) with PC in a cohort of 20 lesions (unresectable primary and metastatic) with oligometastatic presentations [91]. The prescribed dose was 50 Gy/10 Gyfx; the primary endpoint was to deliver adaptive treatment in less than 80 min per fraction for >75% of cases. To meet the planned OAR’s constraints, 75% of the fractions was adapted (mainly for small bowel). Interestingly, a prescribed dose reduction was needed in 43% of cases to stay within the constraints of each session. Moreover, dose escalation over that initially prescribed was possible for three out of 20 patients (none of whom had pancreatic disease). On-line adaptation resulted in Planning Target Volume (PTV) coverage improvement in 57% of cases. Two of the three recurrent pancreatic patients had progression at a median follow-up of 15 months (range 7.5–21 months), while both the primary pancreatic lesion patients were alive without progression at 14 months. No cases of ≥G3 toxicity (sec CTCAE V4) were reported. More recently, a retrospective international multicenter analysis of 44 patients with inoperable pancreatic cancer treated with MRgRT was undertaken by Rudra and colleagues [92]. The study included LAPC, BRPC and medically inoperable patients, treated by different approaches: conventional fractionation (40–55 Gy in 25–28 fractions), hypofractionation (50–67.5 Gy in 10–15 fractions) and SBRT (30–35 Gy in 5 fractions; 40–52 Gy in five fractions). Adaptive MRgRT treatments were delivered to patients receiving 15 or fewer fractions. Patients were stratified into high-dose (biologically effective dose (BED10) > 70 Gy) and standard-dose groups (BED10 ≤ 70 Gy). With a median follow-up of 17 months, BED10 > 70 Gy patients (24; 55%) had significantly improved 2-year OS (49 vs. 30%, *p* = 0.03) compared with the BED10 ≤ 70 Gy patients. Moreover, grade 3+ GI toxicity occurred in three patients in the standard-dose group and did not occur in the high-dose group. These results suggest the potential of MRgRT to help deliver a dose more conformal to the initially planned (since 31 out of 44 patients had been on-line adapted) and confirms the impact of adequate BED on treatment outcome for pancreatic cancer, highlighting the issue for which BED should be preferred for pancreatic cancer [90].

Some technical issues have been highlighted in regard to the complex practical management of on-line adaptive planning with MRgRT. The Dutch group of the Amsterdam UMC first proposed stereotactic MR-guided adaptive radiation therapy (SMART) to focus the daily re-delineation of only the structures of interest into a restricted area within a distance of 3 cm from the PTV surface (SMART3CM) [93]. In their experience, Bohoudi et al. compared 50 previously delivered fractions using the SMART3CM approach, against a simulated standard (re-)planning method through full-scale OAR (re-)contouring (FULLOAR). Plan quality was assessed using PTV coverage (V95%, Dmean, D1cc) and OAR constraints (e.g., V33Gy). PTV coverage was similar using both SMART3CM and FULLOAR (mean V95% = 89%). Adaptive plans using SMART3CM provided lower intermediate and higher doses to all OARs than FULLOAR, which also failed to adhere to the V33Gy dose constraint in 36% of cases. This particular approach is currently used by most of the centers applying on-line-adaptive MRgRT for pancreatic cancer but is not strictly considered the standard. An encouraging evaluation of the clinical effect of SMART was provided by El-Bared et al. [94]. They analyzed 10 non-operated patients with pancreatic cancer (eight with BRPC and two with oligometastatic disease) treated by the SMART3CM approach at 33–40 Gy in five fractions. The dose was prescribed to 90% coverage of the PTV at 100% isodose (PTV100). They compared an adaptive vs. nonadaptive plan of each fraction for each patient. The PTV100 means for adaptive and nonadaptive plans were 90% and 80.4% (range 46–97%), respectively (*p* = 0.0008). Dmax point dose of 38 Gy for duodenum constraint was met in 43 adaptive fractions compared to 32 for nonadaptive fractions (*p* = 0.022). Both PTV100 ≥ 90% and all OAR objectives were achieved in 28 adaptive fractions compared with only three nonadaptive fractions. Bohoudi and colleagues also focused on estimation and prediction of the benefits of the SMART approach for pancreatic cancer [95]. They prospectively collected and analyzed after-treatment data from 36 consecutive LAPC patients. All patients were treated with SMART (40 Gy/5 s) and evaluated in terms of target coverage and OAR sparing in daily plan adaptations. In practice, they compared all the treatment session (180) dose-distribution endpoints for both the baseline (nonadapted) plans and the on-line-adapted plans. They randomly assigned each adapted session as “not needed” (if the original plan already met all constraints), “beneficial” (if the adapted plan provided dosimetrical benefits) or “no benefit” (if the adapted plan failed to provide any benefits). The rate of plans fulfilling constraints increased by 40%, with significant improvements in GTV coverage and lower V33Gy OAR doses. On-line adaptions were “not needed” for 80/180 fractions (44.4%), “beneficial” for 95/180 fractions (52.8%) and of “no benefit” for 5/180 fractions (2.8%). Beside the improvements from half of the sessions, they also found that the improvements were less relevant for presentations with a distance from the tumor to a relevant OAR of >3 mm. An extreme application of the SMART approach was investigated by Lagerwaard et al., looking for intra-fractional modification management alongside that of the inter-fractional by plan adaptation [96]. As the institutional approach of UMC was to deliver SBRT at 40 Gy in five fractions, at three fractions per week, they reported a case of LAPC that received SMART delivered in two split plans each day, allowing for double adaptation each day (i.e., 40 Gy in 10 fractions, with two fractions successively scheduled each day). Both plan re-optimizations appeared important for correcting the inappropriately high duodenal V33 Gy values of 3.6 cc (for the first half baseline) and 3.9 cc (for the second half baseline) to 0.2 cc for both re-optimizations. For the stomach, bowel and all other OARs, high and intermediate doses were well below preset constraints, even without re-optimization. The mean delivery time of each daily treatment was 90 min. Due to the time and resources needed for the process of re-contouring and dosimetric evaluation of a SMART session in PDAC, Tyran and colleagues at University of California, Los Angeles, USA (UCLA) attempted an evaluation of the preliminary image overview by the physician determining the need for starting the adaptation by judging if significant inter-fractional anatomical changes had occurred [97]. Seven patients treated for LAPC or oligometastatic pancreas with SMART at 40 Gy/8 Gyfx, except for one patient who received 33 Gy in five fractions, were included. Thirty-five sets of daily images were analyzed. All fractions retrospectively underwent off-line adaptation: 14/35 fractions were adapted based on overall decisions by physicians compared with 25/35 with off-line reevaluation. Thus, the authors suggested that daily-image visual review is not reliable for determining the benefits of adaptive treatment and that the first step of the SMART procedure (i.e., predicting the daily expected dose distribution through delineation of the daily imaging) seems unavoidable. A multi-institutional prospective trial concerning SMART is ongoing among US Centers (Clinical Trials.gov: NCT03621644). Inclusion of 133 LAPC and BRPC cases is expected, delivering 50 Gy/10 Gyfx (i.e., BED10 = 100 Gy). The primary endpoint is grade 3 or higher acute toxicity (according to CTCAE v5). The restraint of high-dose OAR constraints of a V33 Gy of less than 0.5 cc for the duodenum, stomach and bowel are prioritized on the PTV target coverage. Secondary endpoints include OS, distant progression-free survival and quality of life (QoL). In summary, MRgRT can definitely allow for a safer dose delivery, particularly for SBRT in PDAC, and can allow for safer dose escalation. Whether MRgRT in itself can provide superior results over those of a standard Linac for PDAC has still not been evaluated or reported in the literature, to the best of our knowledge.

### 5.3. Stereotactic Ablative Radiation Therapy (SBRT)

Despite improvements in multimodality approaches combining modern RT techniques, new systemic agents, and surgery, the prognosis of PDAC patients remains unfavorable, regardless of the disease stage [1]. SBRT is a relatively novel option for the treatment of PDAC. According to the ASTRO definition, SBRT is a high-precision image-guided RT technique allowing the delivery of a short course of RT concentrated into between one and five fractions [98]. In fact, high conformality with a rapid dose falloff allows the optimal sparing of the surrounding gastrointestinal (GI) organs while delivering high biologically effective RT doses [99,100]. Advancements in planning, image guidance and delivery are increasingly promoting the use of SBRT in different settings of PC treatment.

#### 5.3.1. Resectable Pancreatic Cancer

To the best of our knowledge, few experiences of SBRT in resectable PDAC have been reported, including two reports published in 2020 [101,102]. The first is a prospective study from a Spanish group [101] including 45 PC patients treated with preoperative CT and SBRT. Only five patients were considered resectable, and four of them underwent radical surgery after CT and SBRT. The second study is a propensity-matched analysis of the National Cancer Database including 2082 patients with resectable PDAC at diagnosis [102]. In this study, 175 patients were treated with neoadjuvant CT + SBRT (median total dose: 35 Gy in 5 fractions) 1355 were treated with CT alone and 552 were treated with CT plus standard external-beam RT. Median OSs were 28, 24 and 23 months in the three groups, respectively (*p* = 0.44). In two-matched comparisons between CT plus SBRT and CT alone (median OS: 30 vs. 21 months, *p* = 0.02), and CT plus SBRT and CT plus external-beam RT, (median OS: 29 vs. 16 months, *p* = 0.002), SBRT plus CT resulted in the most effective combination in term of OS. These results were also confirmed by multivariate analysis. Moreover, SBRT was also associated with a significantly higher pathological complete response rate and higher R0 resections rates compared with the other treatments. Considering that most prospective trials [64,103,104] on preoperative treatment in resectable PDAC have been based on standard conformal RT, future innovative studies based on SBRT techniques seem justified. However, there are no randomized ongoing trials of resectable presentations that investigate the role of SBRT in preoperative settings.

#### 5.3.2. Borderline Resectable Pancreatic Cancer (BRPC)

BRPC is a clinical intermediate stage between resectable and unresectable, even in terms of prognosis. In fact, BRPC, according to the definition of “technical BRPC” [105] is potentially amenable to up-front surgery but has a higher risk of R1 resection and frequent need of vascular resection and reconstruction. According to the American Hepato-Pancreato-Biliary Association classification [106] a BRPC is defined as a tumor with ≤180° abutment around the superior mesenteric artery or vein, celiac axis or portal vein in axial session while, according to the NCCN criteria [3], is a solid tumor with ≤180° contact with celiac axis or >180° without involvement of the aorta or gastroduodenal artery. Instead, “biological BRPC” is defined as a resectable tumor with an unfavorable biology due to poor differentiation, larger tumor size, lymph node metastases and higher CA 19-9 levels [105]. Due to the locally more advanced stage of disease, BRPCs are associated with a significantly worse prognosis compared to resectable presentations. Therefore, there is a strong rationale for BRPC patients to undergo preoperative treatment to improve outcomes [107,108,109,110,111,112,113] and achieves tumor downstaging and downsizing in order to increase the R0 resection rate. Moreover, preoperative treatment produces a therapeutic window of 2–3 months, allowing the diagnosis of early metastatic disease and minimizing the risk of a biologically futile resection. Conversely, neoadjuvant treatment may help the selection of patients with biologically more indolent tumors who will not develop early progressive disease. Nevertheless, most of the evidence regarding neoadjuvant treatment with CT and RT are based on inadequate RT schedules in terms of techniques, volumes, total doses and fractionation [114,115]. Compared to standard RT, SBRT has some theoretical advantages for BRPC, particularly its short duration, and therefore the possibility of avoiding delays or interrupting CT. Moreover, SBRT is able to deliver high BED in few fractions, with high treatment conformality. These characteristics may counteract the intrinsic radiation-resistance of PDAC [116] and may reduce treatment-related toxicity. Nevertheless, robust evidence regarding SBRT in BRPC is lacking. Only a few retrospective studies on SBRT in BRPC were published in the last decade [78,117,118,119,120]. Most of them reported outcomes pooled with other PDAC stages, while only two studies showed a subset analysis in the BRPC setting. Mellon and colleagues [78] treated 110 BRPC patients with CT plus SBRT (30 Gy/5 fractions). Fifty-one percent underwent resection with a 96% R0 resection rate and 19.2- and 34.2-month median OSs for all BRPC patients and resected patients, respectively. Moningi and colleagues [119] treated 14 BRPC patients with CT plus SBRT (25–33 Gy/5 fractions) and reported a 14.4-month median OS with a 29% resection rate. A recent phase I trial on BRPC in SBRT [121] enrolled 13 patients undergoing FOLFIRINOX followed by SBRT. The study defined the maximum tolerated dose of SBRT at the 45 Gy level (36 Gy in 3 fractions to the PTV, with a simultaneous integrated boost to the posterior margin up to 9 Gy in 3 fractions). There were no grade ≥ 3 acute GI toxicities and the median OS and R0 resection rate were 11 months and 66.6%, respectively. For resected patients, median OS was not reached and PFS was 29.6 months. More recently, a phase II trial on SBRT [122] enrolled 18 patients (15 BRPCs and 3 resectable). After 3 cycles of FOLFIRINOX, SBRT was delivered to the tumor and abutting vessel (33 Gy in 5 fractions) with an optional elective PTV (25 Gy in 5 fractions) including lymph nodes and mesenteric vessels. For the entire cohort, this treatment combination was proven to be safe (no ≥grade 3 acute or late GI events) and effective (resection rate: 67%; R0 resection rate: 92%). The median OS and PFS for the entire cohort were 21 and 11 months, respectively. The few BRPC SBRT studies, mainly phase I and phase II trials, had serious enrollment problems as they were unable to reach the initially planned sample size in a reasonable time. This fact discouraged the implementation of randomized trials in this setting. For instance, the NCT 01992705phase I trial on FOLFIRNOX plus SBRT (30 Gy in 5 fractions) in BRPC was discontinued after enrolling only 8 out of the 20 expected participants over 4 years. Moreover, the NCT03099265 phase II trial, based on the same combination but with a higher SBRT dose (33 Gy in 5 fractions), was open for two years but enrolled only 8 out of the 29 expected patients.

A novel SBRT strategy for both BRPC and LAPC is currently being tested by a Chinese group (NCT04289792). The authors expect to enroll 27 patients undergoing split-course SBRT (10 Gy/4 fraction/2 days a week) given as a single 10 Gy fraction on days 2 and 16 of the first two cycles of CT (nab-paclitaxel plus gemcitabine), with PFS and OS as the endpoints. Another ongoing study is the Alliance trial (A021501 - NCT02839343), a multicentric (155 institutions) phase II trial on radiologically, centrally reviewed BRPC patients randomized to receive either eight cycles of FOLFIRINOX or seven cycles of modified FOLFIRINOX followed by SBRT (33–44 Gy in 5 fractions). Thereafter, patients without disease progression will undergo surgery and another four cycles of FOLFIRINOX. The primary endpoint is 18-month OS. At present, 126 out of 134 planned patients have been enrolled.

#### 5.3.3. Locally Advanced Pancreatic Cancer (LAPC)

LAPCs are nonmetastatic but unresectable tumors mainly due to the involvement of adjacent blood vessels. LAPC is a real clinical challenge, being the most common stage at diagnosis (30–40%) [123]. Moreover, although it is well known that mortality from PDAC is mainly caused by distant metastases, two autoptic series [124,125] showed that around 30% of PC patients die due to locally progressive disease alone. Therefore, it can be assumed that an increase of LC rates in LAPC may be a way to improve outcomes in terms of OS, at least in some patient subsets. The previously mentioned advantages of SBRT, as compared to traditional RTCT, could be particularly advantageous in the LAPC setting, particularly in overcoming PDAC intrinsic radioresistance [116] and in promoting optimal integration with CT while minimizing its interruptions or delay [126]. There is a growing body of evidence regarding SBRT in LAPC. For the first time, SBRT for LAPC was investigated at Stanford University in 2004 [127] in a phase I dose escalation trial. The authors delivered up to 25 Gy in a single fraction and reported an 11-month median OS without cases of grade ≥ 3 toxicities. Thereafter, in the same institution, two phase II studies on SBRT (25 Gy in single fraction) delivered using a robotic technique [99] or a standard linear accelerator [128] reported similar median OSs (11.4 and 11.8 months, respectively) but with some cases of severe GI late toxicity. Table 4 summarizes all published studies regarding SBRT for LAPC. After the first pioneering experiments, many institutions tested multi-fraction SBRT regimens often associated with CT instead of single fraction treatments -[100,117,118,119,120,129,130,131,132,133,134]. Besides, these studies showed a good toxicity profile with similar outcomes compared to standard treatments (CT or RTCT). Finally, in 2015, Herman and colleagues [135] performed a phase II trial with the results confirming the positive impact of SBRT in terms of toxicity, OS, LC and pathological complete response. Many recent studies on SBRT +/− CT [78,136,137,138,139] reported outcomes (median OS: 13–19.7 months) (Table 4) comparable with those recorded in two randomized studies, the SCALOP [140] and LAP07 [55] trials, comparing CT vs. CT plus RTCT (median survival: 13.4–16.5 months). Moreover, the systematic review by Petrelli and colleagues [141], including 19 studies with 1009 LAPC patients treated with SBRT, reported a 17-month pooled median OS and 72.3% pooled 1-y LC with late-grade 3–4 toxicity rates not exceeding 11%. The authors also reported a significant correlation of higher total dose (*p* = 0.03) and larger number of fractions (*p* = 0.0019) with improved LC in multivariate random effects model. Similarly, Arcelli and colleagues [136] reported a positive impact of SBRT BEDα/β10Gy ≥ 48 Gy both on LC and OS without increased late GI toxicity rates and the favorable impact of a higher number of fractions on LC through multivariate analysis. On the contrary, the review of Brunner and colleagues [142] showed a negative impact of a higher prescription dose on late toxicity. Particularly, in their analysis, the authors demonstrated an increased incidence of late toxicity while increasing the SBRT dose beyond 75 Gy BED. Based on these results, an emerging approach to improve the results in terms of outcomes while maintaining acceptable toxicity rates is to test dose-escalation modalities based on a higher number of fractions compared to those traditionally used in SBRT [143]. Currently, almost thirty phase I-II trials are ongoing on SBRT for LAPC. Some of them are evaluating the combination of SBRT dose escalation with CT, as is that of an Italian group (NCT03158779) (54 Gy in six fractions plus FOLFIRINOX or gemcitabine-abraxane), or are based on an MRI-LINAC-based approach (NCT03621644).

### 5.4. Simultaneous Integrated Boost (SIB)

The SIB technique is a well-known approach for clinical dose painting and may be used to deliver higher doses to a specific area of the tumor without lengthening the overall treatment time (OTT) [144]. From a radiobiological point of view, this accelerated fractionated strategy allows the increase of the fraction dose and BED to boost volume while the remaining tumor target is covered by a safe, set dose [145]. The use of this tumor burden SIB is currently advocated to improve oncological outcomes in pancreatic cancer in different clinical scenarios. In BRPC (neoadjuvant setting) a selective dose escalation to the tumor-vessel interface (TVI) might improve the likelihood of a negative surgical margin and might reduce the risk of local recurrence. In unresectable LAPC, a SIB dose escalation to the hypoxic center of the pancreatic tumor (potential nest of resistant clones) could improve local tumor control and survival. In addition, for resected patients (up-front or after neoadjuvant chemotherapy), a SIB approach to the surgical bed and/or the positive resection margin (R1) might reduce local recurrence and might improve survival [31,52,122,146].

**Figure 1:**
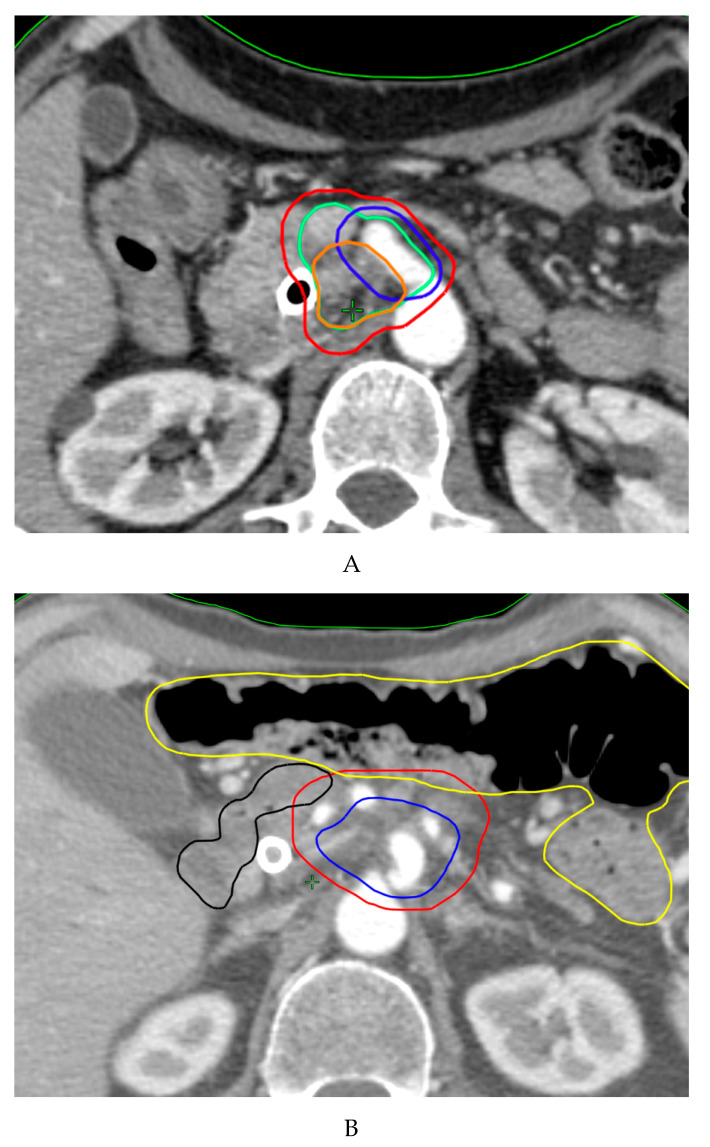
The Simultaneous Integrated Boost (SIB) approach for pancreatic cancer in neoadjuvant (**A**) and definitive (**B**) setting. In BRPC (**A**), the SIB target volume could be directed to the tumor-vessel interface (TVI). PTV tumor = red; PTV high dose (SIB TVI) = blue; CTV = green [GTV (orange) + TVI]. In unresectable LAPC (**B**), the hypoxic center inside the pancreatic tumor could be defined as boost volume. PTV tumor = red; PTV high dose = blue; duodenum = black; bowel = yellow.

**Figure 2:**
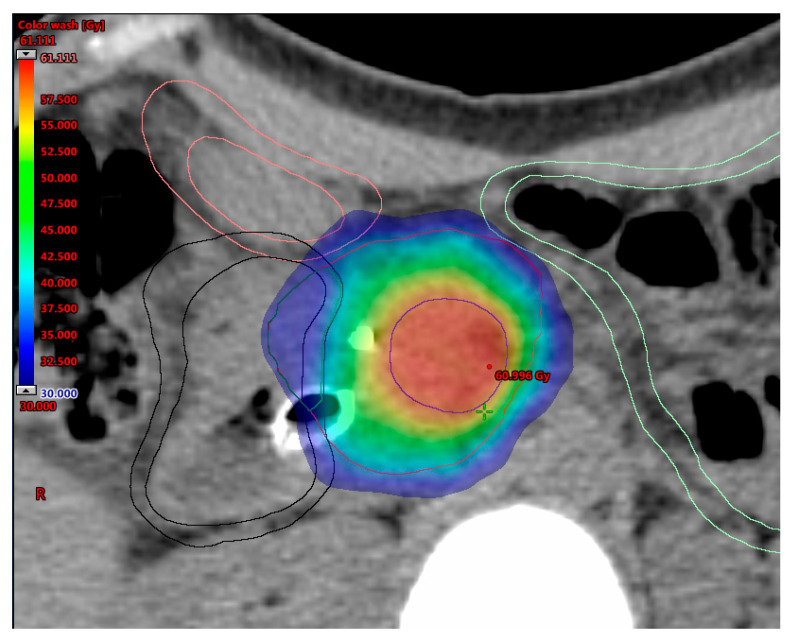
A dosimetric simulation study of the SBRT dose-escalation approach with Simultaneous Integrated Boost (SIB) and Simultaneous Integrated Protection (SIP) for LAPC. This example demonstrates excellent coverage of target volumes and respect of OARs (duodenum = black, bowel = light green, and stomach = pink). The PTV_40Gy_ (red) is created by adding 5 mm to the integrated gross target volume (iGTV) structure. The PTV_60Gy_ (blue) is generated to cover the tumor vessel interface (TVI) inside the iGTV, while the PTV_33Gy_ (dark green) corresponds to the overlap area between the PTV_40Gy_ and PRV OARs (luminal OARs + 3 mm).

Figure 1 outlines possible SIB approaches in pancreatic cancer. Promising data in terms of effectiveness and feasibility of the use of SIB in pancreatic cancer have been shown from heterogeneous protocols with remarkable differences in dose and fractionation (Table 5) [57,78,121,147,148,149,150]. A SIB approach was first described by Chuong et al., supporting the use of SBRT for pancreatic cancer [147]. In an update of their institutional experience, patients suffering from BRPC and LAPC received SBRT delivered in five consecutive daily fractions with a median total dose of 30 Gy to the tumor and a 40 Gy dose painted to the TVI [78]. Median OS was 19.2/15.0 months for BRPC/LAPC patients and increased to 34.2 months in resected patients, with a 1-year local control rate (LRC) of 78% for patients not undergoing resection. A retrospective study evaluating 200 patients with LAPC found that patients receiving dose-escalated IMRT (BED10 > 70 Gy in 15–28 fractions) using a SIB technique had improved OS and locoregional recurrence-free survival compared with standard dose RTCT [57]. Two phase II trials conducted at the Massachusetts General Hospital have recently reported remarkable results of a total neoadjuvant approach in terms of resection rates and oncological outcomes in both BRPC and LAPC patients [149,150]. In these studies, patients with persistent vascular involvement after induction of FOLFIRINOX received long-course (chemo-) radiation therapy (RT) delivered to a dose of 50.4 Gy, with a vascular boost to 58.8 Gy, in 28 fractions. More recently, a practical SIB approach for dose-escalated RT in pancreatic cancer has been proposed by an international panel of radiation oncologists [151]. The authors recommended a SIB up to a BED10 of 100 Gy (ablative dose) by using hypofractionated IMRT (67.5 Gy/15 fractions) or SBRT (50 Gy/5 fractions). However, the prescription of ablative doses is challenging when the tumor is close to critical OARs, such as the duodenum, stomach and bowel, since severe late toxicities (e.g., perforation, stenosis and ulcer with bleeding) are still important concerns in pancreatic cancer RT. In this regard, a new prescription method called simultaneous integrated protection (SIP) has been introduced to prevent toxicity [152]. This is based on the definition of a subvolume as the intersection between the PTV and the planning organ at risk volume (PRVoar), inside which the prescription dose is suitably reduced, according to precise dose constraints. The adoption of SIB with SIP in pancreatic RT may help prevent damage to OARs, thereby enhancing the safe administration of ablative doses to the tumor and maximizing the therapeutic window of clinical benefit [142]. An example of an SBRT SIB/SIP dose escalation approach is shown in Figure 2.

### 5.5. Radiotherapy Combined with Immunotherapy

Despite the success of immunotherapy (IT) in many malignancies, PDAC remains unresponsive to immune checkpoint blockade because of its dense desmoplasia and the phenotype of its immune infiltrate that excludes CD8 T cells. This distinctive microenvironment represents a site of immune privilege [153,154,155,156,157]. Many mechanisms underlie resistance to IT in PDAC including dense stroma that constitutes a physical barrier preventing the delivery of IT into the tumor and trapping T cells, thus preventing their interaction with cancer cells. Particularly, PDAC directly produces stellate cells promoting fibrogenesis through collagen deposition that impairs T cell migration near the tumor. Other tumor microenvironment characteristics play an important role in the immunosuppressive mechanism of PDAC in terms of the production of many cytokines, metabolites and receptors. The latter decrease antigen presentation and support immune-suppressive cell proliferation and inhibition of immune-effective cells [158]. Based on this biological phenotype, non-T cell-inflamed tumors such as PDAC are defined as “cold” and they usually fail to respond to IT.

It has been hypothesized that PDAC immunotherapy resistance can be counteracted through combination with RT, since RT has an immune-modulation effect. In fact, the anticancer effect of RT not only relies on DNA damage but also is based on the interaction with the host immune system, regulating several steps of immune response, such as T cell priming, antigen exposure and presentation [159,160]. Therefore, a novel strategy to overcome the checkpoint blockade resistance of PDAC could be based on the combination of RT and IT. In fact, this may be a way to shift from a “cold” to a “hot” tumor that is amenable to IT.

A recent preclinical study demonstrated that the combination of an agonist CD40 antibody (αCD40) with RT and dual immune checkpoint inhibitors triggers T cells, achieving the aforementioned transformation of a “cold” tumor into a “hot” tumor and therefore generating long term antitumor immunity [161]. Another American group recently showed in a PDAC murine model that the combination of RT, vaccination and αPD-L1 results in improved tumor response, again converting a non-T cell-inflamed to a T cell-inflamed tumor [162]. Moreover, in a preclinical model, the RT upregulation of PD-L1 expression with consequent improvement of antitumor immunity was confirmed [163]. Based on this preliminary evidence, new clinical trials investigating the combination of RT and IT in PDAC seem justified. Actually, the radiosensitizing effect of IT is currently being widely investigated especially in combination with SBRT. As SBRT is usually not delivered with concurrent CT, the RT and IT association could provide an interesting field of study [164]. A phase I/II trial in LAPC has been performed in order to assess safety, efficacy and immunologic correlates of oregovomab (anti–CA-125) followed by SBRT with the radiosensitizer nelfinavir [41]. LAPC patients were initially treated with three cycles of 3 weeks of gemcitabine/leucovorin/fluorouracil/oregovomab. Subsequently, patients received nelfinavir for 5 weeks + concurrent SBRT (40 Gy in 5 fractions). In patients who became resectable after initial treatment, the administration of oregovomab was continued for a further three cycles after surgery. The trial was prematurely closed, and of the 11 patients enrolled, four became resectable. Median OS was 13 months and in the four patients evaluated, and antigen-specific CD8 T-cell immunity was developed due to IT delivery. Finally, at John Hopkins University, the combination of cyclophosphamide plus GVAX plus pembrolizumab and SBRT (33 Gy in 5 fractions) in 50 BRPC patients is currently being tested (NCT03161379). The endpoints of the study, expected to end by 2023, are pathological complete response rate and toxicity.

## 6. Conclusions

Pancreatic cancer represents a major modern oncological challenge and deserves each possible treatment contribution to overcome its aggressiveness. Current standards still rely on the best available options. The best possible standards in new modalities of treatment must be identified. Systemic therapy is, and will remain, a central part of the treatment, but the integration of new modalities with radiotherapy will probably play a crucial role. It is imperative for modern radiotherapy techniques to deliver adequate doses; moreover, clinical trials not investigating such elements should be handled with caution by clinicians. The emerging radio-oncological technologies (particle therapy and MRgRT) and new delivery modality (SIB) will allow revolutionary opportunities. The radiotherapy technique of SBRT will gain significant improvements in both dose delivery and combination with CT to achieve the best outcomes. The integration of radiotherapy with immunotherapy is one of the most promising modern opportunities. Again, the clinical integration of all these opportunities within a multidisciplinary, balanced and focused approach will be determinant in their efficacy.

## Figures and Tables

**Table 1:** Clinical presentation: resectable.

Guideline/Year	International/National (State)	Main Option	Alternative
NCCN 2020 [3]	International	➢ Surgery + Adjuvant Therapy • “Adjuvant Therapy“ includes ○ Clinical Trial (preferred option); ○ CT Alone; ○ CT → RTCT ± CT • Preferred CT Regimens: FOLFIRINOX or mFOLFIRINOX;	• Alternative Adjuvant CT Regimens: Gemcitabine + albumin-bound paclitaxel • Neoadjuvant Therapy for High Risk (includes imaging findings, very highly elevated CA 19-9, large primary tumors, large regional lymph nodes, excessive weight loss, and extreme pain)
ESMO 2015 [21,41]	International	➢ Surgery + Adjuvant CT • CT Regimens: i) gemcitabine or ii) 5-FU folinic acid • Note: No RTCT should be given except in clinical trials	-
PDQ^®^ 2020 [32]	International	➢ Surgery + Adjuvant CT (6 mos) • Preferred CT Regimens: FOLFIRINOX	• Alternative Adjuvant CT Regimens: “Gemcitabine” • Alternative Adjuvant CT Regimens: “5Fu” • Alternative Adjuvant CT Regimens: “S1 in Asia” • Surgery + Adjuvant CT ± RTCT (“controversial”) • Neoadjuvant CT ± RT (“under evaluation”)
ASCO Khoarana 2019 [29]	International	➢ Surgery (“recommended”) + 6-mth adjuvant CT (for: good PS; non extrapancreatic disease; no radiographic interface between primary tumor and mesenteric vasculature; Ca19.9 suggestive of potentially curable disease) • Preferred Adjuvant CT Regimens: mFOLFIRINOX ➢ 6 mos of Neoadjuvant Therapy + Surgery (selected cases)	• Alternative Adjuvant CT Regimens: doublet therapy with gemcitabine and capecitabine or monotherapy with gemcitabine alone or fluorouracil plus folinic acid alone • Adjuvant RTCT after 4–6 mos of adjuvant CT (for R1 and/or N+ patients who have not received Neoadjuvant Therapy • Neoadjuvant Therapy (for good PS; non extrapancreatic disease; no radiographic interface between primary tumor and mesenteric vasculature; and Ca19.9 suggestive of potentially curable disease)
ASTRO 2019 [31]	International	➢ Adjuvant CT alone following R0 resection for pN0 ➢ Adjuvant RTCT following R0 resection for pN+ should be discussed	• Surgery + Adjuvant RTCT for high-risk (R1-R2; pN+) patients is conditionally recommended (4–6 mos after CT) • Surgery + Adjuvant SBRT: only in clinical Trials • Neoadjuvant therapy is conditionally recommended
Hidalgo 2017 [33]	National (Spain)	➢ Surgery + Adjuvant CT (for patients R0/R1; PT1-4/N0-1M0; ECOG PS 0–1; and proper nutritional status)	• “Adjuvant RT” (for R+ and/or N+ patients who did not received preoperatively) • Neoadjuvant Therapy only in Clinical Trials
Neuzillet 2018 [34]	National (French)	➢ Surgery + Adjuvant CT (6 mos; “for all patients”) • Preferred Adjuvant CT Regimens: mFOLFIRINOX	• Alternative Adjuvant CT Regimens: Gemcitabine • Alternative Adjuvant CT Regimens: 5-Fu • Alternative Adjuvant CT Regimens: Gemcitabine+Capecitabine • Adjuvant RTCT is not recommended even in the case of R1 resection (only clinical trials) • Neoadjuvant therapy in Clinical trials
O’Reilly 2018 [35]	National (UK)	➢ Surgery + Adjuvant CT • Preferred Adjuvant CT Regimens: gemcitabine plus capecitabine	• Alternative Adjuvant CT Regimens: Gemcitabine • Unable to make recommendations about adjuvant RTCT • Neoadjuvant therapy only in clinical trials
Seufferlein 2014 - (S3 Guideline) [36]	National (German)	➢ Surgery + Adjuvant CT (6 mos; also for R0/R1 resection) • Preferred Adjuvant CT Regimens: gemcitabine or 5-fluorouracil (5-FU)	• Adjuvant RTCT only in randomized controlled trials • “Neoadjuvant RT, RTCT or CT only in randomized controlled trials
Yamaguchi 2017 [37]	National (Japan)	➢ Surgery + Adjuvant CT • Preferred Adjuvant CT Regimens: “S-1 monotherapy”	• Alternative Adjuvant CT Regimens: gemcitabine hydrochloride monotherapy • Adjuvant RTCT is not recommended • IORT is not recommended • Neoadjuvant therapy (“CT or RTCT”) only in clinical trials
AIOM 2019 [39]	National (Italian)	➢ Surgery + Adjuvant CT; (also for R0/R1 resection) • Preferred Adjuvant CT Regimens: “FOLFIRINOX”	• Alternative Adjuvant CT Regimens: “Gemcitabine+Capecitabine”; for R0/R1 resection • Alternative Adjuvant CT Regimens: “Gemcitabine” for 6 mos; for R0/R1 resection • Alternative Adjuvant CT Regimens: “5FU/Leucovorin” for 6 mos; for R0/R1 resection • Surgery + Adjuvant CT (“Capecitabine” for 6 mos → RTCT; for selected patients) • Neoadjuvant CT→Surgery →Postoperative CT (3+3 mos)
Hyde 2019 [38]	National (Eastern Canada) Consensus Conference	➢ Surgery + Adjuvant CT (6 mos; “for all stages”) • Preferred Adjuvant CT Regimens: “mFOLFIRINOX”	• Alternative Adjuvant CT Regimens: “Gemcitabine-Capecitabine” (6 mos; “for all stages”) • Alternative Adjuvant CT Regimens: “Gemcitabine” (6 mos; “for all stages”) • Neoadjuvant CT (under investigation; to be considered) • Neoadjuvant RT (under investigation; to be considered) • “Superiority of preoperative RTCT over preoperative CT has not been unequivocally demonstrated” • “RT could be considered in high-risk disease”

Legend: CT: Chemotherapy; RTCT: Radiochemotherapy; RT: Radiotherapy; SBRT: sterotactic RT; IORT: Intraoperative RT; BRT: Brachitherapy; GTV: Gross Tumor Volume; CTV: Clinical Target Volume; GEM: Gemcitabine; 5-Fu: 5_Fluoruracil; Lnf: lymph-nodes; mo: months; R0: Microsopically negative resection; R1: Microsopically positive resection; pN+: pathologically positive nodal status; M0: absence of distant metastases; ➢ Primary indication; • Details reported; ○ Alternatives (if present) for the same priority level/detail indicated.

**Table 2:** Clinical presentation: Locally Advanced Pancreatic Cancer (LAPC).

Guideline/Year	International/National (State)	Main Option	Alternative
NCCN 2020 [3]	International	➢ Clinical Trial (preferred option); • CT Alone; • CT (4–6 mos)→ RTCT or SBRT (for selected patients) • RTCT • SBRT (for selected patients) • Preferred CT Regimens: FOLFIRINOX or mFOLFIRINOX;	• Alternative Adjuvant CT Regimens: Gemcitabine + albumin-bound paclitaxel • Alternative Adjuvant CT Regimens: Gemcitabine • Alternative Adjuvant CT Regimens: Capecitabine • Alternative Adjuvant CT Regimens: Continuous infusion 5-FU • Palliative therapy
ESMO 2015 [28,42]	International	➢ CT (6 mos) • Preferred CT Regimens: Gemcitabine	• RTCT + Capecitabine (minor role)
PDQ^®^ 2020 [32]	International	➢ CT • Preferred CT Regimens: “FOLFIRINOX” • Preferred CT Regimens: “nab-paclitaxel/gemcitabine”	• Alternative Adjuvant CT Regimens: “gemcitabine” • RTCT (“controversial”) • CT Novel Agents ± RTCT (“under clinical evaluation”) • IORT (“under clinical evaluation”) • BRT (“under clinical evaluation”)
ASCO Balaban 2017 [30]	International	➢ CT (for PS ECOG 0-1; favorable comorbidity profile) • Preferred CT Regimens: no clear evidence to support one regimen	• Upfront RTCT or SBRT (“on the basis of patient and physician preference”) • RTCT or SBRT after 6 mos of CT (if response or stable disease) • RTCT or SBRT in the case of local only progression
ASTRO 2019 [31]	International	➢ If LAPC is selected for possible downstaging: • CT → RTCT (Conditional recommendation; 4–6+ mos after CT) • CT → SBRT multifraction (Conditional recommendation) ➢ If LAPC is NOT possible for downstaging • CT → RTCT (4–6+ mos after CT) • CT → RTCT (dose escalation) • CT → SBRT multifraction	-
Hidalgo 2017 [33]	National (Spain)	➢ CT (3–4 mos reassessment) → Surgery or CT ± RT (for partial response and stable disease) • Preferred CT Regimens: “FOLFIRINOX” • Preferred CT Regimens: “GEM-nab-paclitaxel”	For pt candidates for CT with limitations: Alternative Adjuvant CT Regimens: CT (single drug) Alternative Adjuvant CT Regimens: CT (double drug) “RT alone”
Neuzillet 2018 [34]	National (French)	➢ CT • Preferred CT Regimens: Gemcitabine • Preferred CT Regimens: “FOLFIRINOX” • Preferred CT Regimens: GEM-nab-paclitaxel	• RTCT (capecitabine) after CT if “Tumor Control”
O’Reilly 2018 [35]	National (UK)	➢ CT • Preferred CT Regimens: not to make a specific recommendation (Gemcitabine/FOLFIRINOX allowed)	• Alternative Adjuvant CT Regimens: Gemcitabine alone (for pt unlikely to tolerate combination therapy) • Unable for specific recommendation on the use of consolidation RTCT (anyway to be preferred with capecitabine) • Irreversible electroporation only in research context
Seufferlein 2014 - (S3 Guideline) [36]	National (German)	➢ CT	• CT →RTCT (if stable disease)
Yamaguchi 2017 [37]	National (Japan)	➢ RTCT (“with fluoropyrimidine or gemcitabine hydrochloride”; “3DRT”; “CTV= GTV+Lnf showing frequent metastases”) ➢ CT (“until progression”) • Preferred CT Regimens: “Gemcitabine hydrochloride monotherapy”, • Preferred CT Regimens: “S-1 monotherapy, Preferred CT Regimens: “FOLFIRINOX • Preferred CT Regimens: “Gemcitabine hydrochloride + Nab-paclitaxel”) ➢ CT → RTCT ➢ IORT	• Alternative Adjuvant CT Regimens: Gemcitabine hy- drochloride + S-1
AIOM 2019 [39]	National (Italian)	➢ CT (Authors state “Very Low Quality of Evidence”) • Preferred CT Regimens: “Gemcitabine” • Preferred CT Regimens: “FOLFIRINOX • Preferred CT Regimens: PEXG/PAXG • Preferred CT Regimens: “Gemcitabine + Abraxane)	• CT → RTCT (capecitabine-based; for patientsECOG<2; M0)
Hyde 2019 [38]	National (Eastern Canada) Consensus Conference	➢ Issue not expressly addressed	• CT + RTCT (“could be considered for high risk patients”; “aim of local control improvement”; “should be delivered using modern techniques”; optimal dose to be defined) • SBRT (in clinical trial)
van Veldhuisen 2019 [40]	National (Dutch)	➢ CT (4–6 mos) • Preferred CT Regimens: “Gemcitabine” • Preferred CT Regimens: “FOLFIRINOX • Preferred CT Regimens: PEXG/PAXG • Preferred CT Regimens: “Gemcitabine + Abraxane)	• After CT: if Progression→CT • After CT: if Non-Progression, Inoperable→Local ablation ○ Irreversible Electroporation ○ Radiofrequency Ablation ○ SBRT • After CT: if Non-Progression, Operable→CT

Legend: e-update availabe at ESMO web-site; Available online: URL https://www.esmo.org/guidelines/gastrointestinal-cancers/pancreatic-cancer/eupdate-cancer-of-the-pancreas-treatment-recommendations. (accessed on 16 June 2020) CT: Chemotherapy; RTCT: Radiochemotherapy; RT: Radiotherapy; SBRT: sterotactic RT; IORT: Intraoperative RT; BRT: Brachitherapy; GTV: Gross Tumor Volume; CTV: Clinical Target Volume; GEM: Gemcitabine; 5-Fu: 5_Fluoruracil; Lnf: lymph-nodes; mo: months; R0: Microsopically negative resection; R1: Microsopically positive resection; pN+: pathologically positive nodal status; M0: absence of distant metastases; ➢ Primary indication • Details reported ○ Alternatives (if present) for the same priority level/detail indicated.

**Table 3:** Clinical presentation: Borderline Resectable Pancreatic Cancer (BRPC).

Guideline/Year	International/National (State)	Main Option	Alternative
NCCN 2020 [3]	International	➢ Neoadjuvant Therapy → Evaluation for Surgery Neoadjuvant Therapy Includes: • CT ± subsequent RTCT ○ Preferred CT Regimens: FOLFIRINOX or mFOLFIRINOX; ○ Preferred CT Regimens: Gemcitabine ± albumin-bound paclitaxel • RTCT	• Only for known BRCA1/2 or PALB2 mutations: ○ FOLFIRINOX or mFOLFIRINOX ± subsequent RTCT ○ Gemcitabine + cisplatin (≥2–6 cycles) ± subsequent RTCT • Adjuvant Therapy for Resected patients
ESMO Ducreux 2015 [28,42]	International	• Clinical Trials (“*wherever possible”*) • CT→RTCT→ Evaluation for Surgery (“*In routine practice”*) • Preferred CT Regimens: Gemcitabine • Preferred CT Regimens: FOLFIRINOX	-
PDQ^®^ 2020 [32]	International	➢ Neoadjuvant CT ± RT → Evaluation for Surgery	• Preoperative CT ± RT • Preoperative RT • Alternative RT techniques
ASCO [29]	International	Not expressly specified: see “resectable”	-
ASTRO 2019 [31]	International	Conditional recommendation for: ➢ CT → RTCT (2–6 mos after CT) ➢ CT → SBRT (multifraction; 2–6 mos after CT)	-
Hidalgo 2017 [33]	National (Spain)	➢ Neoadjuvant Therapy Includes: • CT→ Multidisciplinary reassessment (3–4 mos) ○ Preferred CT Regimens: Gemcitabine-nab-paclitaxel ○ Preferred CT Regimens: FOLFIRINOX • RTCT (either 5-Fu or GEM; IMRT encouraged)	-
Neuzillet 2018 [34]	National (French)	• Clinical Trials (“*wherever possible”*) • Neoadjuvant CT ± RTCT • Preferred CT Regimens: FOLFIRINOX • Preferred CT Regimens: Gemcitabine- nab-paclitaxel	-
O’Reilly 2018 [35]	National (UK)	• Clinical Trials	-
Seufferlein 2014 -(S3 Guideline) [36]	National (German)	Not specifically addressed	-
Yamaguchi 2017 [37]	National (Japan)	• Clinical Trials • “preoperative treatment improves resection rate of the surgical resection and an R0 rate and may be connected to the improvement of the clinical outcome”	-
AIOM 2019 [39]	National (Italian)	➢ Neoadjuvant CT → Evaluation for Surgery • Preferred CT Regimens: “Gemcitabine” • Preferred CT Regimens: “FOLFIRINOX • Preferred CT Regimens: PEXG/PAXG • Preferred CT Regimens: “Gemcitabine + Abraxane)	• After Neoadjuvant CT: if Local Progression→RTCT ± CT • After Neoadjuvant CT: if Stable or Responsive disease →Surgery ± CT ± RT
Hyde 2019 [38]	National (Eastern Canada) Consensus Conference	➢ Neoadjuvant Therapy → Evaluation for Surgery Includes • Neoadjuvant CT →RTCT (for non progressive patients after CT; RTCT questionable if patients is operable after CT; RT by VMAT or IMRT is preferable; RTCT optimal dosing and delivery have yet to be determined) ○ “Superiority of preoperative RTCT over preoperative CT has not been unequivocally demonstrated”	• Neoadjuvant SBRT in clinical trials

Legend: e-update availabe at ESMO web-site; Available online: URL https://www.esmo.org/guidelines/gastrointestinal-cancers/pancreatic-cancer/eupdate-cancer-of-the-pancreas-treatment-recommendations. (accessed on 16 June 2020) CT: Chemotherapy; RTCT: Radiochemotherapy; RT: Radiotherapy; SBRT: sterotactic RT; IORT: Intraoperative RT; BRT: Brachitherapy; GTV: Gross Tumor Volume; CTV: Clinical Target Volume; GEM: Gemcitabine; 5-Fu: 5_Fluoruracil; Lnf: lymph-nodes; mos: months; R0: Microsopically negative resection; R1: Microsopically positive resection; pN+: pathologically positive nodal status; M0: absence of distant metastases; ➢ Primary indication; • Details reported; ○ Alternatives (if present) for the same priority level/detail indicated.

**Table 4:** Studies on stereotactic body radiotherapy in borderline resectable and locally advanced pancreatic cancer.

Author, Year	Enrolment Period	Study Design	Study Sample	N° BRPC	N° LAPC	% of Patients Treated with Total Dose (Gy) and Fractionation (N°)	Technique	Pre-SBRT CT (%)	Total Median OS (Months)	BRPC Median OS (Months)	LAPC Median OS (Months)	Total Resectios (%)	BRPC Resection (%)	LAPC Resection (%)	Total R0 Resections (%)	BRPC R0 Resection (%)	LAPC R0 Resection (%)	Late GI Toxicity Grade (G): (%)
Arcelli, 2020 [136]	2013-2018	Retr	56		56	18–45/3–5	VMAT IMRT RS 3-D	55.3			19.0							G ≥3: 2.5
Kharofa, 2019 [122]	2014–2017	Ph II	18	15		44%: 33/5 56%: 33/5 + 25/5 (SIB)		mFolfirinox: 100.0	21.0			67.0			92.0			G ≥3: 0
Jung, 2019 [137]	2011–2016	Retr	95		95	24–36/4	VMAT IMRT	Gem-based: 10.0 Folfirinox: 3.2			16.7			7.4			3.2	G ≥3: 3.2
Chapman, 2018 [138]	2012–2016	Retr	75	53	22	24–40/NR		Folfirinox: 64.0 Gem-based:34.7 Other: 1.3		23.5	19.7		81.6	7.0				
Ryan, 2018 [139]	2010–2016	Retr	29			25–33/5					13.0							G ≥3: 4
Mellon, 2015 [78]	2009–2014	Retr	159	110	49	28–30/5	IMRT	Gem: 86.0 Folfirinox: 14.0	18.1	19.2	15.0	38.0	51.0	14.0	38.3	96.0	10.0	G ≥3: 7
Shaib, 2016 [121]	2011–2015	Ph I	13	12		25%: 30/3 + 6/3 (SIB) 25%: 36/3 + 6/3 (SIB) 25%: 36/3 + 7.5/3 (SIB) 25%: 36/3 + 9/3 (SIB)	VMAT (SIB)	mFolfirinox: 100.0		11.0			66.6			66.6		G ≥3: 0
Moningi, 2015 [119]	2010–2014	Retr	88	14	74	25–33/5	NR	88.0	18.4	18.4	14.4	21.6	28.5	20.2	84.0			G ≥3: 1.1
Herman, 2015 [135]	2010–2012	Ph II	49		49	33/5	VMAT-IMRT	Gem: 90.0			13.9			8.0		8.0		G ≥2: 11
Song, 2015 [129]	2006–2014	Retr	59		59	35–50/3–5–8	RS				12.5							G ≥3: 2
Pollom, 2014 [120]	2002–2013	Retr	167	11	133	45%: 25/1 55%: 25–45/>1	VMAT	87.5										
Tozzi, 2013 [130]	2010–2011	Retr	30		21	83%: 45/6 17%: 36/6	VMAT	30.0	11.0									G ≥3: 0
Rajagopalan, 2013 [118]	2008–2011	Retr	12	7	5	58%: 36/3 42%: 24/1	RS	91.7	47.2						91.7			G ≥3: 0
Gurka, 2013 [100]	2009–2011	Ph I	10		10	25/5	RS	Gem: 100.0			12.2							G ≥3: 0
Boone, 2013 [117]	2011–2012	Retr	9	4	5	36/3	RS	100.0					75.0	20.0		50.0		
Goyal, 2012 [131]	2007–2010	Retr	19		19	74%: 20–25/1 26%: 24–30/3	RS	68.0			14.3							G ≥3: 16
Schellenberg, 2011 [128]	2006–2007	Ph II	20		20	25/1	IMRT	Gem: 100			11.8							G ≥3: 5
Polistina, 2010 [132]	2004–2007	Ph II	23		23	30/3	RS	Gem: 100			10.6	8.0						G ≥3: 0
Rwigema, 2011 [133]	2004–2009	Retr	71		40	18–25/1–3	RS		10.3		6.2							G ≥3: 0
Mahadevan, 2011 [134]	2007–2010	Retr	47		39	71.7%: 24/3 28.3%: 30/3	RS	Gem: 100			20.0							G ≥3: 9
Schellenberg, 2008 [128]	2004–2006	Ph II	16		16	25/1	RS	Gem: 100		11.4								G ≥3: 12.5
Koong, 2004 [127]	2001–2006	Retr	15		15	20.0%: 15/1 33.3%: 20/1 46.7%: 25/1	RS			11.0								G ≥3: 0

BRPC: borderline resectable pancreatic cancer; Gem: gemcitabine; GI: gastrointestinal; IMRT intensity modulated radiotherapy; LAPC: locally advanced pancreatic cancer; mFOLFIRINOX: modified FOLFIRINOX; OS: overall survival; Ph: phase; Retr: retrospective; RS: radiosurgery SBRT: stereotactic body radiotherapy; VMAT: volumetric modulated arc therapy; 3-D: three-dimensional.

**Table 5:** Recent findings on the application of SIB on pancreatic cancer (summary).

Study, Year [ref]	Study Type	Tumor Stage	Fractions (n) Tumor Target Definition		Tumor Target Dose (Gy)	SIB Target	SIB Dose (Gy)	Study Primary Endpoint	Late Toxicity G ≥ 3
Chuong, 2013 [147]	Retrospective	BRPC/LAPC	5	PTV = entire tumor + 3–5 mm	25	TVI (region of vessel abutment/encasement)	35	OS (m): 16.4/15 PFS (m): 9.7/9.8 BRPC/LAPC	5.3%
Passoni, 2013 [148]	Phase II	LAPC	15	PTV = ITV (tumor and enlarged lymph nodes plus motion) + BTV + 5/7 mm	44.25	Infiltrating vessel + 1 cm within GTV	48-58	DLT: not reached	0%
Mellon, 2015 [78]	Retrospective	BRPC/LAPC	5	PTV = GTV (plus motion) + 3–5 mm	30	TVI (areas of vessel involvement by tumor)	40	OS (m): 19.2/15 LCR: 78% *	7%
Krishnan, 2016 [8]	Retrospective	LAPC	28 15	PTV = GTV + 15 mm	50.4 37.5	GTV + 2–5 mm	63–70 67.5	OS (m): 17.8 PFS (m): 10.2	No additional compared to SDR
Shaib, 2016 [121]	Phase I	LAPC	3	PTV = GTV with at-risk area of microscopic spread + 5 mm	12	PM = volume between the posterior 1 cm of GTV and mesenteric vessel/retroperitoneal soft tissue	15	DLT: not reached	0%
Murphy, 2018 [149]	Phase II	BRPC	28	PTV = CTV (GTV + 1 cm margin and elective nodal coverage) + 7 mm	50.4	TVI (tumor involvement of critical blood vessels)	58.8	R0 resection rate: 97%	0%
Murphy, 2019 [150]	Phase II	LAPC	28	PTV = CTV (GTV + 1 cm margin and elective nodal coverage) + 7 mm	50.4	TVI (tumor involvement of critical blood vessels)	58.8	R0 resection rate: 81%	0%

SIB = Simultaneous Integrated Boost, n = number, Gy = gray, G = grade, BRPC = borderline resectable pancreatic cancer, LAPC = locally advanced pancreatic cancer, PTV = planning target volume, TVI = tumor-vessel interface, OS = overall survival, m = months, PFS = progression-free survival, BTV = biological tumor volume, DLT = dose-limiting toxicity, CTV = clinical target volume, LCR = local control rate, GTV = gross tumor volume, SDR = standard dose radiation therapy, PM = posterior margin. * patients not undergoing resection.
